# Examining the Impact of Service Quality on Passengers’ Intentions to Utilize Rail Transport in the Post-Pandemic Era: An Integrated Approach of SERVQUAL and Health Belief Model

**DOI:** 10.3390/bs13100789

**Published:** 2023-09-22

**Authors:** Panuwat Wisutwattanasak, Thanapong Champahom, Sajjakaj Jomnonkwao, Fareeda Aryuyo, Chamroeun Se, Vatanavongs Ratanavaraha

**Affiliations:** 1 Institute of Research and Development, Suranaree University of Technology, Nakhon Ratchasima 30000, Thailand; panuwat.w@g.sut.ac.th (P.W.); fareeda@sut.ac.th (F.A.); chamroeun.s@g.sut.ac.th (C.S.); 2 Department of Management, Faculty of Business Administration, Rajamangala University of Technology Isan, Nakhon Ratchasima 30000, Thailand; thanapong.ch@rmuti.ac.th; 3 School of Transportation Engineering, Institute of Engineering, Suranaree University of Technology, Nakhon Ratchasima 30000, Thailand; vatanavongs@g.sut.ac.th

**Keywords:** COVID-19, railway service quality, structural equation modeling, confirmatory factor analysis, post-epidemic

## Abstract

The COVID-19 pandemic has significantly disrupted railway transportation in developing countries, resulting in reduced passenger demand and economic activity. As a result of the pandemic effect, there is an increased focus on health and safety among potential passengers. To address this issue, the present study aimed to investigate the fundamental factors that contribute to railway transportation service quality (SERVQUAL) and the intention to use intercity train services in Thailand using the health belief model (HBM), with 1600 passenger participants using structural equation modeling (SEM). The findings demonstrated that train operators’ service standards remain vital to passengers, and the HBM had a statistically significant impact on shaping passengers’ intentions to use train services after an epidemic. These results can inform rail agencies and health authorities when developing policies and strategies to prioritize both business and passenger safety on intercity trains.

## 1. Introduction

### 1.1. Background Problem and Location

The transportation system plays a crucial role in facilitating the movement of people and goods from one place to another. Its significance extends beyond mere convenience, as it contributes substantially to a country’s social and economic development by connecting its various systems. As populations grow and societies expand, the demand for transportation-related aspects, such as travel and the transport of goods, increases. However, without an efficient transportation system in place, a country’s ability to meet these demands and support its social and economic systems becomes limited. Therefore, it is imperative to develop a transportation system that can effectively respond to the needs of its users. In terms of mode choices, railway transportation stands out as the oldest and most cost-effective means of land transit, offering a more sustainable option. This makes it particularly suitable for low- and middle-income individuals [1,2]. However, certain countries’ rail transport systems have yet to meet passengers’ expectations, especially in developing nations [3,4]. Enhancing the quality of train services would not only benefit low- and middle-income users but also improve the overall quality of life for a significant portion of the population.

Thailand, classified as a developing country within the low- and middle-income group, holds significant importance as a research location. Situated in the heart of Southeast Asia (SEA), Thailand serves as an exemplary representation of the tourism perspective and requirements within this region. However, the quality of railway transportation has unfortunately fallen short of passengers’ expectations. Over the past three decades, Thailand has primarily focused on the development of road transportation, as highlighted by the Asian Development Bank [5]. Consequently, the railway network remains significantly smaller in comparison to the extensive road network. Nevertheless, considering the objective of sustainable transportation, it is crucial to reduce reliance on road transportation due to the increased likelihood of accidents and higher associated costs when compared to other modes of transportation [6]. Thus, Thailand stands as a suitable candidate to represent developing nations, ultimately serving as a guiding light for the enhancement of rail transport service quality.

### 1.2. Rail Transportation Context after the Large Epidemic

Nevertheless, the development of a strategy in the service dimension alone may not be sufficient to meet the expectations of current users. The COVID-19 pandemic has caused a shift in users’ perspectives, leading them to prioritize their health status and hygiene [7,8,9]. In the post-pandemic period, public transport must implement stringent health protocols in its operations to ensure safety [10]. The global impact of the COVID-19 pandemic has been detrimental to all countries, resulting in significant political, social, transportation, and economic slowdowns worldwide [11]. Although the circumstances vary across nations, there have been substantial declines in public transportation usage recorded during the pandemic. In Hong Kong, for instance, 40% of survey respondents stated that they would avoid using public transportation as a precautionary measure during the early stages of the epidemic [12]. A study conducted in Turkey also revealed that avoiding public transportation was one of the most commonly adopted preventive measures during the pandemic [13]. Similarly, in Sweden, where the public was encouraged to work from home whenever possible and use public transportation only when absolutely necessary, research by Jenelius and Cebecauer [14] found a 60% reduction in public transportation trips in Stockholm and a 40% reduction in Västra Götaland. Budapest, Hungary experienced an increase in car usage from 43% to 65% following the implementation of COVID-19 travel restrictions, while public transportation utilization dropped from 43% to 18% [15]. Thailand’s public transportation has also suffered significant consequences as the tourism and service sectors, which contribute to the majority of the country’s domestic income and account for 52.4% of GDP [16], are severely affected. Existing literature [12,13] suggests that safety and health considerations significantly influence users’ intentions to utilize public transport.

Research on rail transport service in the post-pandemic era remains an indispensable and highly relevant area of investigation, underpinned by a multitude of compelling reasons. While past research efforts have indeed addressed the enhancement of rail transport service quality, including studies conducted both before [17,18,19] and during the COVID-19 pandemic [10,20], the transformative impact of the pandemic on various facets of daily life, particularly in the realm of transportation, underscores the distinct and pressing need for further examination in this context. The challenges confronting rail transport services in the post-pandemic period are notably distinct from those encountered in preceding times, demanding a nuanced understanding and tailored responses. These challenges encompass dynamic shifts in passenger expectations, the emergence of new safety protocols, and fundamental alterations in travel behaviors. Of paramount significance is the evolution of passenger preferences and priorities in direct response to the pandemic’s far-reaching effects. Safety, hygiene, and health considerations have ascended to the forefront of passenger concerns [7,21], necessitating astute comprehension and effective accommodation by rail transport providers.

In essence, the imperative for research in this domain transcends the boundaries of historical studies. While prior investigations have indeed yielded valuable insights into the quality of train services, the unique post-pandemic landscape presents a distinctive set of imperatives and opportunities. As such, this study focusing on post-pandemic railway service quality assumes a pivotal role in bridging this consequential gap in research. By offering empirical evidence and informed perspectives, it contributes substantively to the ongoing pursuit of railway service improvement, which is relevant across temporal eras.

### 1.3. Related Theories in Public Transport Service Research

In the context of research concerning railway service quality, several studies have shown that psychological frameworks, such as the theory of planned behavior (TPB) and the technology acceptance model (TAM), can be applied to understand public transit service quality perceptions. Recent research, as exemplified by works of Donald et al. [22], Chen and Chao [23], and Mann and Abraham [24], has indicated that the TPB is significantly correlated with service quality research and public transport use. As such, the TPB stands out as one of the most pivotal concepts in studying customers’ perceptions of service quality. Amid the backdrop of the COVID-19 pandemic, the emphasis on passengers’ perspectives, awareness of the virus, and concerns about health and safety have become central in the travel industry. As underscored in the pertinent literature [7,8,10,25] it is crucial for service providers to address these facets. Even though the acute phase of the pandemic has subsided and COVID-19 has been reclassified as an endemic disease, one cannot overlook the sustained awareness among users regarding the perceived severity and concerns of infection. In early 2023, studies by Ding et al. [26] and Hidayat and Choocharukul [10] highlighted that public transport users still hold expectations regarding healthcare and safety management. These studies found a robust correlation between these concerns and users’ perceptions of and intentions to use public transport. Nevertheless, despite these concerns, there is a lack of research applying psychological and health perspectives to the analysis of railway transport service quality. By adopting this approach, we can gain valuable insights into passengers’ behavioral patterns and preferences regarding rail transportation usage post-COVID-19. Consequently, this study seeks to incorporate psychological theories pertaining to healthcare and safety alongside service quality indicators to evaluate users’ intentions towards using rail transportation. The health belief model (HBM)—a theory deeply rooted in health concerns and encompassing various psychological components like perceived susceptibility, perceived severity, perceived barriers, perceived benefits, cues to action, and health motivation—is poised to offer valuable insights into users’ behavior. Employing this model can elucidate the perspectives and attitudes of health-conscious individuals regarding travel safety under unique circumstances, such as a post-pandemic world. It is crucial to note that this research does not aim to discredit the well-established findings of the TPB in service quality research. Instead, it seeks to broaden the research domain by introducing healthcare and safety dimensions through the HBM, an approach not previously utilized in railway service quality studies in a post-pandemic context.

### 1.4. Research Gap and Objectives

**Figure 1:**
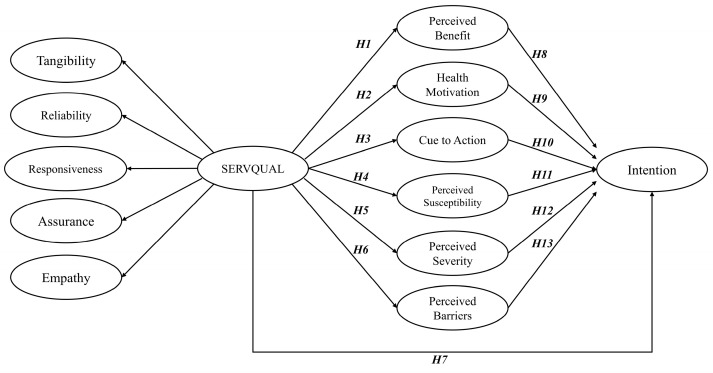
Conceptualization of the proposed model.

Nowadays, psychological theories have been applied in diverse ways within the realm of public transport studies. However, the utilization of the HBM in the context of transportation research, particularly concerning rail transport service quality, remains underexplored. This study aims to shed light on this gap in the literature by demonstrating that healthcare and safety management will emerge as pivotal concerns for users in the post-pandemic era. Additionally, it is worth noting that there has been no prior investigation into the synergistic relationship between the SERVQUAL model and the HBM in rail transportation. Exploring the interplay between these two conceptual frameworks holds the potential to unveil essential expectations and perspectives regarding healthcare and safety during transportation usage (the conceptual model will be proposed in Section 3.1 (Figure 1)). These insights may significantly impact individuals’ intentions to utilize the public transport system.

To address existing gaps in the literature, this study aims to examine the interaction between the HBM and SERVQUAL. It seeks to investigate the factors that influence the intention to use intercity rail transport, particularly in the context of a developing country during the post-pandemic period. This study employs confirmatory factor analysis (CFA) and structural equation modeling (SEM), which are econometric analyses that are suitable for the analysis of questionnaire research. The study results can be divided into two main points: (1) identifying the expectations of railway passengers regarding service quality after an epidemic, and (2) determining the components of the HBM that influence passengers’ intentions to use rail transportation. This study makes a significant contribution by affirming the enduring importance of traditional service quality components, as encapsulated in the SERVQUAL framework, even in the post-pandemic era. Furthermore, this study extends the boundaries of research on the determinants of service quality expectations, by encompassing dimensions related to perceptions of healthcare and safety. These findings can provide relevant authorities with guidance on how to improve their strategies to attract passengers and encourage their intentions to use the service in a post-pandemic context.

The remainder of this study is organized as follows: Section 2 provides a comprehensive literature review and the research hypotheses, while Section 3 presents the research methodologies employed, including the questionnaire structure, data collection, and analysis method. In Section 4, the results of the statistical analysis are presented and discussed. Finally, the concluding section summarizes the key findings of the study and outlines potential implementation strategies.

## 2. Literature Review

This section presents a comprehensive literature review that examines the factors influencing users’ intentions to use railway transportation. The review primarily focuses on two key aspects: the HBM and indicators of railway transport service quality. These factors were carefully examined to gain deeper insights into their impact on user behavior and preferences.

### 2.1. Theory of Health Belief Model

The HBM is a psychological theory that pertains to health management and proves to be highly relevant when studying rail transport service quality. Given the post-pandemic era, tourists now place greater emphasis on health-related factors. Consequently, adopting the HBM concept allows us to understand the attitudes and viewpoints of travelers who prioritize their health while using railway transportation. As a psychosocial model of health behavior change, the HBM enables the description and prediction of health-related behaviors [27]. According to this model, individuals are more likely to seek guidance and adhere to recommendations for prevention and rehabilitation if the preventive measures are relatively easy to implement. In essence, the HBM encompasses beliefs, feelings, thoughts, and understanding pertaining to recognizing and accepting one’s health conditions. The HBM consists of six key factors: perceived susceptibility, perceived severity, perceived barriers, perceived benefits, cues to action, and health motivation. These components are interconnected and influence individual behaviors from a health perspective. Therefore, it can be hypothesized that users who possess strong beliefs and awareness regarding health will demonstrate an intention to respond to various factors in order to achieve and maintain good health.

The literature review reveals that previous studies on the HBM predominantly focused on road safety and health-related domains. For instance, Razmara et al. [28] and Morowatisharifabad [29] found that the HBM served as the most reliable predictor of safe driving behavior. Similarly, Rezapur-Shahkolai et al. [30] examined the impact of the HBM on preventive behaviors related to road traffic injuries and observed that the HBM could enhance health awareness and injury prevention performance. Moreover, Zhang et al. [31] demonstrated that the HBM could explain behavior associated with injuries, particularly in the context of traffic accidents. However, it is worth noting that the majority of these studies focused on factors associated with unsafe driving behavior, thus neglecting investigations into passengers’ intentions to use rail transportation services through the lens of the HBM. Addressing this research gap, the current study aims to utilize the HBM to understand passengers’ thoughts and awareness regarding their health status, which in turn would influence their intention to utilize rail services in the context of the post-pandemic.

### 2.2. SERVQUAL

The concept of service quality development, initially introduced by Parasuraman et al. [32], evolved significantly over time. In its initial phase, Parasuraman et al. [32] proposed a comprehensive set of ten determinants that underpinned perceived service quality. These elements encompassed access, communication, competence, courtesy, credibility, reliability, responsiveness, security, tangibles, and understanding/knowing the customer. After a careful review, research found that some of these dimensions were highly correlated and could be consolidated into broader categories. This led to the refinement of the SERVQUAL model, reducing the ten elements to five essential dimensions, as proposed by Parasuraman et al.’s [33] second-phase model. These dimensions were found to capture the key aspects of service quality and were more manageable and practical for measuring and evaluating service quality effectively. The revised model has since been widely adopted and utilized in various service industries to assess and improve service quality [20,34]. This study recognizes the importance of SERVQUAL as having the potential to improve service quality in all dimensions and aims to apply this conceptual framework, in conjunction with the health belief model (HBM), to investigate the factors that influence rail transport service. In the following sections, we will outline these five elements and elucidate their relevance to the context of rail transport service, particularly in light of the ongoing COVID-19 situation.

#### 2.2.1. Tangibility

The tangibility of a business encompasses the appearance of its physical facilities, equipment, staff, and communication materials. Customers hold certain expectations in terms of cleanliness and professionalism when it comes to facilities and stores, as well as a preference for well-groomed and neatly presented workers. This aligns with the findings of Miranda et al. [17] and Gopal Vasanthi et al. [21], whose research corroborates a strong and positive correlation between tangibility and passenger satisfaction. The study conducted by Sama et al. [20] further underscores the increased relevance of the tangibility dimension, particularly during the COVID-19 situation. Visible and tangible signs of safety measures, such as sanitization stations, physical distancing markers, and personal protective equipment (PPE) availability, contribute to passengers’ confidence in the rail service’s efforts to ensure a safe travel experience.

#### 2.2.2. Reliability

Reliability refers to the consistent and accurate performance of promised services. It is crucial for organizations to refrain from deceiving customers and instead deliver on their claims. Customer satisfaction holds immense importance, as customers seek the assurance that your business will provide functional products or effective services, offer timely assistance when needed, and consistently meet their expectations. Wan et al. [35] explained that reliability elements of public transport would help to improve service performance. In the COVID-19 situation, reliability takes on an essential role in the rail transport service. Passengers need assurance that the rail service will operate consistently and predictably during these challenging times. This includes adherence to schedules, providing timely information about any service changes or disruptions due to the pandemic, and ensuring that safety measures are consistently implemented to safeguard passengers’ health.

#### 2.2.3. Responsiveness

Responsiveness entails demonstrating a proactive approach in assisting customers and delivering prompt service. In today’s fast-paced society, it is crucial to respond swiftly to consumer inquiries and concerns. This principle applies even when clients may be slow in their own responses. By promptly acknowledging their requests and communicating that you are actively working on resolving them, you provide assurance and maintain a sense of responsiveness towards your customers. Huang et al. [18] and Sama et al. [20] reported that responsiveness is an important measure of quality in train transportation. Responsiveness is crucial during the COVID-19 situation as it pertains to handling passengers’ inquiries, concerns, and feedback promptly and effectively. Timely responses to safety-related queries, service updates, and health-related issues can significantly impact passengers’ perception of the rail service’s dedication to their well-being.

#### 2.2.4. Assurance

Assurance encompasses the knowledge, courtesy, and capability of employees in conveying trust and confidence to customers. Customers have an expectation for service providers to possess expertise in their respective fields. By effectively communicating your proficiency to customers through various means such as displaying credentials, industry certifications, or sharing customer testimonials, you reinforce their trust in your business. Nguyen-Phuoc et al. [19] work reported that providing a safe public transportation environment is critical in shaping passengers’ loyalty intentions because people are exposed to critical incidents throughout the service delivery, whether during access or egress, waiting at transit terminals or stops, or onboard [36]. The pandemic has heightened passengers’ concerns about safety and well-being during travel. Assurance becomes crucial in demonstrating the rail service’s commitment to providing a secure and reliable environment. This can be achieved through clear communication about the safety measures in place, training staff to handle health-related inquiries, and showcasing the implementation of best practices to minimize the risk of COVID-19 transmission.

#### 2.2.5. Empathy

Empathy refers to the provision of caring and personalized attention by a firm towards its customers. Customers desire to feel valued beyond mere transactions and aim to establish a meaningful relationship with your business. Demonstrating empathy involves actively showcasing your company’s genuine concern for their well-being. By training employees on delivering exceptional and empathetic service, where warm smiles and engaging conversations are frequent occurrences, you can surpass customer expectations [21]. Hamzah et al.’s [36] findings indicated that empathy is the specific service quality component of primary importance and performance, increasing passengers’ satisfaction. The pandemic has brought forth a wide range of emotional responses from passengers, including fear and anxiety. Demonstrating empathy becomes crucial for rail transport service providers. This involves understanding passengers’ concerns, addressing their questions and worries, and showing compassion towards any difficulties they may face while traveling during the pandemic.

Overall, the five elements of the SERVQUAL model are interconnected and play a vital role in shaping passengers’ experiences during the COVID-19 situation in rail transport service. By addressing these dimensions and adapting their service approach accordingly, rail service providers can foster trust, enhance customer satisfaction, and ensure the safety and well-being of their passengers amidst the ongoing pandemic.

## 3. Methods

### 3.1. Study Design

Figure 1 illustrates the proposed model, which aims to examine the correlation between intercity train service quality and rail passenger intention, with factors related to the HBM playing a mediator role.

The utilization of the SERVQUAL model indicators to predict rail service quality is not a new concept. For example, Parasuraman et al. [32] demonstrated that SERVQUAL, measured through dimensions like tangibility, reliability, responsiveness, assurance, and empathy, significantly predicted service quality in the hospitality sector. Hence, it is logical to suggest that these indicators can serve as components of SERVQUAL.

In the aftermath of the widespread COVID-19 pandemic, global transportation passengers have grown increasingly concerned about health awareness [8,10]. Consequently, the significance of service quality and goods safety has a direct positive impact on various aspects, including the perceived benefits, cues to action, and motivation related to using these services. However, the emergence of a perceived barrier as a negative aspect, indicating resistance to change (specifically regarding the use of rail transportation during the epidemic), is a significant concern. Consequently, a higher perception of service quality leads to reduced infection concerns and subsequently results in a decrease in perceived barriers (suggesting a negative correlation). Moreover, the perceived threat, encompassing susceptibility and severity, aligns with the perceived barrier. Wu and Chiang’s [37] research demonstrates that respondents who were aware of their vulnerability and the severity of COVID-19 were more inclined to seek vaccination due to infection concerns. Conversely, in the context of this study, if service providers implement effective measures and improve service quality, the concern for COVID-19 when using rail transport diminishes (leading to a decrease in the perceived threat). In light of this context, the study aims to investigate the positive and negative influence of the SERVQUAL model on key factors of the HBM, which are as follows:

**Hypothesis 1 (H1).**  *SERVQUAL has a positive effect on perceived benefits.*

**Hypothesis 2 (H2).**  *SERVQUAL has a positive effect on health motivation.*

**Hypothesis 3 (H3).**  *SERVQUAL has a positive effect on cue to action.*

**Hypothesis 4 (H4).**  *SERVQUAL has a negative effect on perceived susceptibility.*

**Hypothesis 5 (H5).**  *SERVQUAL has a negative effect on perceived severity.*

**Hypothesis 6 (H6).**  *SERVQUAL has a negative effect on perceived barriers.*

The HBM components have been extensively studied, as demonstrated by previous research conducted by Huang et al. [18] and Kim et al. [38]. Huang et al. [18] investigated the relationship between health beliefs, self-efficacy, preventive behaviors, and travel satisfaction among individuals traveling to high-altitude destinations. The study found that perceived benefits are essential antecedent beliefs in attitudes towards preventive behaviors. Another study conducted by Hüsser et al. [39] applied the HBM framework to predict tourists’ willingness to apply non-pharmaceutical interventions (NPIs) against COVID-19 while traveling. The study revealed that attitudes play a crucial role in determining behavioral intentions to apply NPIs while traveling, with the aforementioned studies having demonstrated a positive association between perceived benefit, cue to action, and health motivation with behavioral intention. Furthermore, a positive correlation is observed between good SERVQUAL and passengers’ intentions to use rail transportation services. Building on these findings, hypotheses were formulated concerning passengers’ intentions to utilize rail transportation services, incorporating elements from both the HBM and SERVQUAL.

**Hypothesis 7 (H7).**  *SERVQUAL has a positive influence on intention.*

**Hypothesis 8 (H8).**  *Perceived benefit has a positive influence on intention.*

**Hypothesis 9 (H9).**  *Health motivation has a positive influence on intention.*

**Hypothesis 10 (H10).**  *Cue to action has a positive influence on intention.*

On the other hand, Razmara et al. [28] found that perceived barriers exhibit a negative correlation with behavioral intention. The inherent tendency of humans to develop routines for repetitive actions and their fear of change can act as obstacles to accepting new behaviors, as observed in the context of the COVID-19 situation. Unusual factors can lead passengers to worry and feel insecure about using the service. Additionally, Morowatisharifabad [29] revealed a negative correlation between perceived threat (susceptibility and severity) and behavior, with these factors being associated with health concerns. This implies that if respondents perceive themselves as vulnerable or anticipate severe consequences of COVID-19 during rail transport use, they are less likely to intend to use the service. Conversely, if respondents have lower levels of concern about such issues, their intention to use rail transport will likely increase. Based on these insights, the hypotheses for the remaining constructs can be formulated as follows:

**Hypothesis 11 (H11).**  *Perceived susceptibility has a negative influence on intention.*

**Hypothesis 12 (H12).**  *Perceived severity has a negative influence on intention.*

**Hypothesis 13 (H13).**  *Perceived barrier has a negative influence on intention.*

### 3.2. Setting

In the context of this specific study, the sample comprised 1600 intercity rail passengers hailing from four distinct routes within Thailand, namely the North, Northeast, East, and South routes, each of which was represented by 400 respondents. To gather survey data, data collection took place at intercity train terminals, totaling 32 terminals in all, with 8 terminals allocated to each route. The selection of train stations as survey sites was advantageous for efficient data collection. This approach was beneficial because the questionnaire included inquiries relevant to both the station and the train. The survey was administered during the months of June and July in the year 2023. Notably, the questionnaire was designed to be efficient, requiring no more than 15 min for each respondent to complete.

### 3.3. Participants

The criteria for participant inclusion in this study revolved around individuals who have utilized intercity rail transportation both during past pandemics and in the post-pandemic COVID-19 era. This criterion was established to ensure that participants possessed a genuine inclination to provide relevant information and harbored a sincere concern regarding the potential implications of future pandemics on public transportation. In striving for a representative sample, the study made concerted efforts to encompass participants that mirror the diversity of the overall population. This inclusivity was achieved through the consideration of various demographic factors, including gender, age, education level, and income, among others, when selecting and recruiting participants. Table 1 presents the preliminary statistics of the respondents (the reliability of the questionnaire ranged between 0.726 and 0.883.).

### 3.4. Variables

In this study, we established pertinent variables by adapting component indicators from the health belief model (HBM) and SERVQUAL model used in previous research. These variables were then re-contextualized within the context of analyzing factors that influence the intention to utilize rail transport services. The latent variable within the SERVQUAL model encompasses five essential elements: tangibility, reliability, responsiveness, assurance, and empathy, constituting a total of 20 observed indicators, as presented in Table 2. Similarly, our analysis considered 24 observed indicators representing the various components of the health belief model (HBM), including perceived susceptibility, perceived severity, perceived barriers, perceived benefits, cues to action, and health motivation. In addition to these, the outcome of this study focused on identifying relevant indicators capable of predicting passengers’ intentions to use rail transport services within the framework of a post-pandemic scenario. This outcome comprised four observed indicators strategically chosen for analysis.

### 3.5. Data Sources and Measurement

The research questionnaire is structured into three sections to gather relevant information. The first section focuses on sociodemographic details and travel experiences. The second section explores various factors that influence the quality of railway transport service, encompassing tangibility, reliability, responsiveness, assurance, and empathy. Lastly, the third section investigates factors pertaining to the HBM. The context of these questions relates to the quality of service for both regular travel and potential scenarios involving epidemics in the future. Section 2 and Section 3 of the questionnaire utilize a 7-point Likert scale [40], with higher scores indicating stronger agreement (7) or disagreement (1), and these were approved by the Index of Item-Objective Congruence test of three service quality research specialists. The questionnaire items and descriptions are provided in Table 2.

**Table 2:** Questionnaire items.

Items	Description	Adapted From
	SERVQUAL	
	Tangibility;	[17,21]
TAN1	Railway employees have clear and correct communications, even in unusual situations.	
TAN2	Timetables, display boards, etc. are eye-catching, even in unusual situations.	
TAN3	The train stations and their toilets are kept clean, even under unusual circumstances.	
TAN4	Staff are cleanly uniformed and polite in every situation.	
	Reliability;	[17,21,34]
REL1	The train arrives and leaves on time in all situations.	
REL2	Provide fair service and do not take advantage of passengers or users.	
REL3	When there is a problem, the railway staff shows sincerity by solving the problem for you.	
REL4	The train never broke down during the journey.	
	Responsiveness;	[17,34]
RES1	Staff are happy to help immediately.	
RES2	The staff is available for service and changes. With advance communication.	
RES3	The train staff are there to respond or assist you even when you are busy.	
RES4	Staff provide timely and efficient service.	
	Assurance;	[21]
ASS1	Traveling by train makes me feel safe, even under unusual circumstances.	
ASS2	Railway employees are courteous in service.	
ASS3	Employees have in-depth training and knowledge.	
ASS4	The behavior of staff builds confidence in passengers.	
	Empathy;	[17,21]
EMP1	Employees are attentive individually, whether or not problems arise in every situation.	
EMP2	Train travel is convenient for all passengers, such as children, the elderly, the disabled, and pregnant women.	
EMP3	The service provider always considers the best interests of passengers as a priority.	
EMP4	Rail operators make it easy to plan your trip.	
	Health belief model	
INT	Intention;	[41]
INT1	I intend to continue using the train in any situation.	
INT2	I will recommend to my family that they use rail transportation.	
INT3	I will recommend to the people around me that they use the rail transportation service.	
INT4	I always plan to take the train whenever possible.	
PSU	Perceived Susceptibility;	[41,42]
PSU1	I’ve heard of viral outbreaks or diseases affecting train users.	
PSU2	I will feel uncomfortable when traveling by train during the epidemic.	
PSU3	There is a chance that my image will be damaged when taking the train during the epidemic.	
PSU4	I know that every time I use rail transportation, I am at risk of contracting the plague.	
PSE	Perceived Severity;	[42,43]
PSE1	I know that if I get an infection or an epidemic while traveling on the train, it might make me sick.	
PSE2	Infection from an epidemic can result in disability or death.	
PSE3	Sickness from an infection will greatly affect your studies and work.	
PSE4	Each illness or death is a waste of time and money for me and my family.	
PSE5	Illness and death affect the lives of people I know, such as my family.	
PBE	Perceived Benefits;	[41,43]
PBE1	I think traveling by train will be more beneficial to me than driving a personal car, even during the pandemic.	
PBE2	I think traveling by train can give me more value than other modes, despite the pandemic.	
PBE3	I feel that taking the train will keep me safe, even in unusual situations.	
PBE4	I think train travel can help reduce the chances of infection or epidemic disease.	
PBA	Perceived Barriers;	[13,43]
PBA1	I feel uncomfortable when taking trains in unusual or pandemic situations.	
PBA2	I feel like a freak when using the train, while others have ceased to use the service.	
PBA3	Despite the unusual situation, the train service has not yet been stopped. I will still be able to use rail transportation.	
PBA4	I feel that the unusual situation will make using the train more difficult.	
CUE	Cues to Action; (0.824)	[44]
CUE1	I have many friends who regularly take the train in any situation.	
CUE2	My residence has easy access to rail transport, so I use it regularly in all situations.	
CUE3	People around me have used rail transportation regularly since I was a child.	
CUE4	I regularly get compliments when using rail transportation as an alternative to transportation.	
MOT	Health Motivation; (0.726)	[45]
MOT1	I value the safety of my family’s life and property.	
MOT2	I thought that if I caught an epidemic from train travel, it would be the worst.	
MOT3	I think using rail transport is a safe and cost-effective form of travel in all situations.	

### 3.6. Bias

This study recognizes the potential sources of bias that could have arisen during the research process, specifically focusing on the distribution of samples and participants’ understanding of the detailed questionnaire items. To effectively address these concerns, a comprehensive approach was adopted. The study opted for face-to-face interviews conducted by academic staff, a strategy aimed at ensuring that respondents thoroughly comprehended the research proposal and the nuances of the questionnaire. Furthermore, in a concerted effort to minimize distribution bias, the study took deliberate measures to survey a sample that was as evenly distributed as possible, as elaborated in the previous section. These steps were taken to enhance the validity and reliability of the study’s findings, thereby mitigating potential sources of bias.

### 3.7. Study Size

Golob (2003) introduced sampling methods tailored for structural equation modeling. These methods are grounded in the principle that the sample size used for estimating maximum probability should ideally be at least 15 times the number of observable variables (Pituch and Stevens, 2015). Applying this guideline to our study, which encompasses 50 observed variables, a minimum sample size of 15 times 50, equivalent to a sample of 750, is necessitated. The process of sample selection for this study meticulously adhered to these requirements for SEM analysis. Furthermore, our study sought to ensure comprehensive coverage across all four routes. Consequently, the data collection effort was structured to encompass 400 participants per route, resulting in a cumulative total of 1600 participants. It is essential to underscore that this sample size was strategically determined to meet the statistical prerequisites for analysis.

### 3.8. Statistical Methods

#### 3.8.1. Confirmatory Factor Analysis and Structural Equation Modeling

To confirm the correlations among the components derived from the SERVQUAL [32] and HBM [27], we employed confirmatory factor analysis (CFA). CFA was initially developed by Jöreskog [46] and serves to ascertain the consistency of measures with the scholarly comprehension of associated factors. The primary objective of CFA is to evaluate the extent to which the collected data align with the research hypotheses [47].

In the data analysis, SEM was implemented due to its theoretical foundation that emphasizes the relationship between latent variables and observable variables. SEM utilizes both a measurement model and a structural model to establish the causal links between variables [48,49]. To assess the suitability of the data for the SEM approach, various statistical techniques were employed, including factor analysis, path analysis, and regression models. Such methods will be analyzed using Mplus 7.2 software. These techniques collectively enable a comprehensive evaluation of the data within the SEM framework.

To assess the suitability of the component data, we subjected each indicator’s model results to statistical testing. For this purpose, we utilized the criteria proposed by Fornell and Larcker [50] and Hair [51], which involve evaluating the average variance extracted (*AVE*) and composite reliability (*CR*) values. For a valid fit, the *AVE* and *CR* values should both exceed 0.5 and 0.7, respectively. These statistical values can be calculated using Equations (1) and (2) as follows:

(1) $$AVE=\frac{\sum_{i=1}^{n}\lambda_{i}^{2}}{n},$$

(2) $$CR=\frac{{(\sum_{i=1}^{n}\lambda_{i})}^{2}}{{(\sum_{i=1}^{n}\lambda_{i})}^{2}+(\sum_{c=1}^{n}\delta_{i})}$$

where $\lambda_{i}$ denotes the component loading of each indicator, and $\delta_{i}$ represents the error terms.

Based on the CFA results, this study proceeds with SEM to analyze the data’s structural aspects and the path effects among SERVQUAL, the HBM, and passengers’ intentions to use rail transportation. Before conducting SEM analysis, it is crucial to verify that there are no excessively high correlations between pairs of components (i.e., the main factors of SERVQUAL and the HBM). To address this concern, we utilize the Pearson correlation matrix, as a high correlation might introduce model bias. Once we pass the CFA and correlation test, we move on to employing SEM to analyze the overall model structure and measurements, as presented in Figure 1 (previous figure).

#### 3.8.2. Model Fit Criteria

In order to validate the correctness and model fit, the analysis must take into account several statistical values that assess the ability of the model to explain the relationships under investigation. The most common hypothesis test in SEM is the chi-square test, which compares the observed and expected covariance matrices under the model. The chi-square test has a null hypothesis that the model fits the data perfectly and an alternative hypothesis that the model does not fit the data. A low chi-square value indicates a good fit, while a high chi-square value indicates a poor fit. These values include the chi-square per degree of freedom $\left(\chi^{2}/df\right)$, which ideally should not exceed 5 in the initial evaluation [52,53]. Additionally, the root mean square error of approximation (RMSEA) presents how well the model, with unknown but optimally chosen parameter estimates, would fit the populations covariance matrix [48]. In recent years it has become regarded as ‘one of the most informative fit indices’ due to its sensitivity to the number of estimated parameters in the model. The recommendation for RMSEA is that the cut-off point should be below 0.07 to indicate a good fit [54]. Another indicator of model fit is the Tucker–Lewis index (TLI), which is also called the non-normed fit index (NNFI) and ranges between 0 and 1, with larger values indicating better relative fit. The TLI measures a relative reduction in misfit per degree of freedom. This index was originally proposed by Tucker and Lewis [55] in the context of exploratory factor analysis and later generalized to the covariance structure analysis context. Previous research recommended that the suitable TLI should be equal to or greater than 0.80 [56]. The comparative fit index (CFI) is the statistic that assesses the model by comparing the $\chi^{2}$ value of the model to the $\chi^{2}$ of the null model. The null/independence model is the worst-case scenario as it specifies that all measured variables are uncorrelated. This statistic assumes that all latent variables are uncorrelated (null/independence model) and compares the sample covariance matrix with this null model. Recent studies have shown that a value greater than 0.90 is needed in order to ensure that misspecified models are not accepted [57], while the standardized root mean square residual (*SRMR*) is the square root of the difference between the residuals of the sample covariance matrix and the hypothesized covariance model. The values for the *SRMR* range from zero to 1.0; however, values as high as 0.08 are deemed acceptable (Hu and Bentler, 1999). A *SRMR* of 0 indicates perfect fit but it must be noted that the *SRMR* will be lower when there is a high number of parameters in the model, and in models based on large sample sizes [57,58]. These statistical testing values can be calculated using Equations (3)–(6), as outlined below:

(3) $$SRMR=\sqrt{\sum_{i}\sum_{k}\frac{r_{jk}}{p*}},$$

(4) $$RMSEA=\sqrt{\frac{\chi_{T}^{2}-{df}_{T}}{{df}_{T}(N-1)}},$$

(5) $$TLI=1-\frac{max[\left(\chi_{T}^{2}-{df}_{T}\right),0]}{max[\left(\chi_{T}^{2}-{df}_{T}\right),\left(\chi_{B}^{2}-{df}_{B}\right),0]},$$

(6) $$CFI=\frac{\left(\frac{\chi_{B}^{2}}{{df}_{B}}\right)-\left(\frac{\chi_{T}^{2}}{{df}_{T}}\right)}{\left(\frac{\chi_{B}^{2}}{{df}_{B}}\right)-1}.$$

When $r_{jk}$ is the standardized residuals from a covariance matrix with *j* rows and *k* columns, and p∗ is the number of non-duplicated elements in the covariance matrix.$\;\chi_{T}^{2}-\chi^{2}$ are the values of the target model, ${df}_{T}=df$ is the target model, $\chi_{B}^{2}-\chi^{2}$ are the values of the baseline model, and ${df}_{B}=df$ is the baseline model.

## 4. Results and Discussion

### 4.1. Observed Indicators and Descriptive Statistics

Table 3 presents the descriptive statistics derived from the questionnaire responses for the two models: SERVQUAL and HBM. The provided statistics encompass measures such as mean, standard deviation, skewness, kurtosis, and reliability tests. It is noteworthy that we examined the descriptive statistics to assess the normal distribution, adhering to Kline [47]’s recommendation that skewness should fall within the range of −2 to 2, and kurtosis between −7 and 7. In line with Tavakol and Dennick’s [59] assertion, adequate reliability is indicated when the Cronbach’s alpha value is 0.7 or higher. Significantly, the statistical values obtained in this study were found to be within acceptable ranges for analysis.

### 4.2. Analysis of Factors Affecting Passengers’ Intentions to Use Rail Transport

#### 4.2.1. Measurement Indicators of the HBM and the SERVQUAL Model

In this section, we employed CFA to establish the representativeness of observed indicators derived from rail transport passengers in relation to the latent factors, i.e., SERVQUAL and the HBM. The CFA results were obtained using Muthén and Muthén’s Mplus 7.2 software. Our analysis revealed that all indicators significantly contributed to the SERVQUAL and the HBM factors, with each parameter showing significance at the 0.01 level. While the appropriate AVE and CR values for all factors in this study met the criteria of 0.5 and 0.7, respectively, it is essential to consider the findings from previous literature [50,60]. Their research confirmed that when CR exceeds 0.7, an AVE value higher than 0.4 is still considered acceptable. Therefore, our results align with the acceptable threshold as per the established literature. The model fit statistics (see table footnote) indicate a good fit between the model and empirical data, meeting the acceptance criteria. For further details on the model estimation results, please refer to Table 4.

Regarding the table, all indicators obtained from research questionnaires can be used as components of the HBM and SERVQUAL factors with 0.01 level significance. The results illustrated that INT3 (“I will recommend to the people around me that they use the rail transportation service”) is the most explainable factor for explaining passengers’ intention. Thus, PSU1 (“I’ve heard of viral outbreaks or diseases affecting train users”) plays the role of the most influential factors as a component of perceived susceptibility. Next, perceived severity found that PSE5 (“Illness and death affect the lives of people I know, such as my family”) was the best representative indicator. The perceived benefit revealed that PBE2 (“I think traveling by train can give me more value than other modes, despite the pandemic”) was the most influential indicator for explaining users’ views. Furthermore, the perceived barrier had indicator PBA3 as the most representative factor (“Despite the unusual situation, the train service has not yet been stopped. I will still be able to use rail transportation”). Then, cue to action was derived as CUE2, “My residence has easy access to rail transport, so I use it regularly in all situations”, was the highest measurement indicator. Finally, for the health motivation of users, it was found that MOT1 (“I value the safety of my family’s life and property”) was the most influential thing that passengers value.

For the concept of SERVQUAL, the factor that was a good fit for tangibility is TAN3: “The train stations and their toilets are kept clean, even under unusual circumstances”. The results revealed that most passengers have focused on cleanliness and hygiene as affected by the epidemic. Second, the reliability of rail service was best measured by REL3: “When there is a problem, the railway staff shows sincerity by solving the problem for you”. Next, it was also found that RES4 (“Staff provide timely and efficient service”) was the most important indicator for responsiveness of service from the perspective of rail users. Further, ASS3, “Employees have in-depth training and knowledge”, was the most influential factor in representing the assurance of rail transportation. Lastly, service empathy could be measured the most by EMP1: “Employees are attentive individually, whether or not problems arise in every situation”.

Regarding Table 4, we ensured the absence of high correlations (multicollinearity) among the main factors of the model by conducting correlation tests. The beta weights were used to compute all observed indicators into the main factors, resulting in five constructs for the SERVQUAL model: tangibility, reliability, responsiveness, assurance, and empathy. Additionally, the HBM model comprised seven constructs: perceived susceptibility, perceived severity, perceived benefit, perceived barriers, cue to action, health motivation, and intention. The correlations between these constructs are presented in Table 5.

To assess the discriminant validity, we examined the square roots of the AVE, which provide a good explanation of the constructs. Prior studies have suggested that the square roots of AVE for each factor should be greater than the correlation coefficients of their counterparts [51,61]. Our results confirmed that the statistical values fall within the acceptable range. Furthermore, according to Mukaka [62], correlations between relevant variables should be less than 0.8, which we also found to hold true in our study. These findings provide strong evidence of the validity and reliability of the model.

#### 4.2.2. Influence of the HBM on Service Quality Expectation

**Figure 2:**
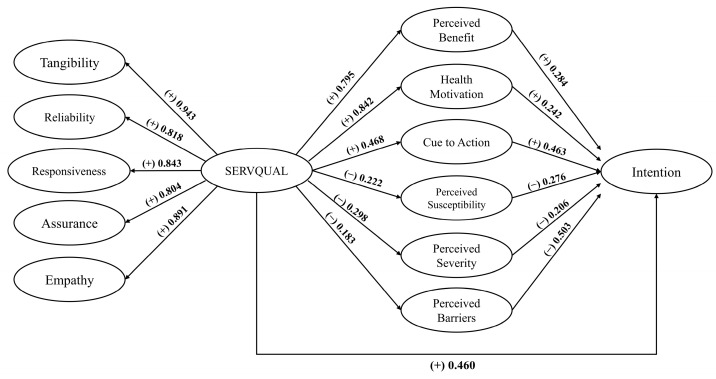
Model estimation results of passengers’ intentions to use rail transportation.

Table 6 and Figure 2 display the outcomes of the model estimation concerning the determinants impacting passengers’ inclination to utilize rail transportation within a novel travel epoch. These determinants were initially postulated in the conceptual model. The goodness of fit of the model is evidenced by the following statistics: $\chi^{2}/df$ = 2.795; CFI = 966; TLI = 960; SRMR = 0.038; and RMSEA = 0.033. The practical model integrates indicators derived from SERVQUAL and incorporates the HBM to elucidate the behavioral intent associated with railway utilization. The ensuing sections will furnish comprehensive elaboration on each facet.

Regarding the findings, they underscore and validate the enduring significance of the SERVQUAL model components in explicating the service quality inherent to rail transportation. Notably, “tangibility” surfaces as the foremost influencer, an emblematic indicator constituting a pivotal facet of service quality as perceived by passengers. This concurs with the conclusions of Miranda et al. [17] and Gopal Vasanthi et al. [21], whose investigations substantiate the affirmative and substantial correlation between tangibility and passenger contentment. Subsequently, “empathy” takes the stage, encapsulating considerations of attentiveness and deference toward passengers’ interests. As such, empathy emerges as an indispensable cornerstone of railway service, aligning with the insights of Gopal Vasanthi et al. [21], which accentuate the need for a robust emphasis on empathy for the enhancement of service quality from the passengers’ vantage. Furthermore, the pivotal role of “responsiveness” in gauging service quality finds validation, a stance corroborated by the findings of Shi et al. [34]. Moving forward, the facet of “reliability” comes to the fore, embodying customer trust and conviction nurtured through organizational and service reliability—facets such as punctuality, impartiality, and efficient grievance redressal. These factors engender passenger confidence and foster an enduring rapport with the service, an insight echoed by [34]. Additionally, “assurance”, the final strand within the SERVQUAL framework, while exhibiting comparably lower factor loading, retains its significance within the passenger paradigm. Its pertinence is evident from the work of Hundal and Kumar [63], whose findings underscore the significant associations between all SERVQUAL indicators and the perceived service quality among intercity rail passengers.

Building upon the findings regarding hypotheses **H1**, **H2**, and **H3**, this study delved further into the pivotal role that the level of service quality required by passengers can play in positively influencing factors such as perceived benefits, health motivation, and cues to action for the post-pandemic era. Based on this premise, a clear conclusion can be drawn: enhancing the quality of service provision would lead passengers to perceive the benefits and value of the service more profoundly. The correlation between health motivation and perceived benefit is evident. When passengers recognize that a service contributes to an improved quality of life or provides safety and healthcare support, which is particularly relevant during epidemics, their motivation for maintaining health is amplified. Cues to action encompass the impact of human trends, ease of accessibility, and advertising. Rail transportation services that manifest significant improvements in service quality, such as emphasizing the importance and robustness of their offerings or ensuring convenient access, tend to generate greater incentives for passengers to opt for these services.

Furthermore, the outcomes stemming from **H4**, **H5**, and **H6** provide substantiated support for the adverse impact of SERVQUAL on perceived susceptibility, perceived severity, and perceived barriers within the context of the study. Within the framework of a post-pandemic scenario, the notion of perceived threat is intricately linked to the risk and severity associated with COVID-19 infection. Consequently, these concerns tend to escalate when the quality of rail transport services diminishes. As such, the enhancement of rail service quality assumes a crucial role in serving as an indicator for mitigating risks. Ding et al.’s [26] findings demonstrated that improving service quality (such as cleaning public transport vehicles and stations) is also an important component of mitigating passengers’ risk perceptions.

Furthermore, it is worth noting that SERVQUAL exerts a negative influence on perceived barriers. The sweeping global changes triggered by significant events such as pandemics elicit heightened apprehensions among passengers regarding unforeseen incidents. Navigating public transportation becomes an arduous task in the midst of a pandemic, primarily due to the pervasive fear of epidemics acting as a formidable barrier for passengers. Hence, the amelioration of service quality emerges as a potent strategy that bolsters passenger confidence and subsequently diminishes these barriers.

In the context of factors positively shaping passengers’ intentions to utilize railway transportation, as evidenced by **H10**, the results incontrovertibly demonstrate that cues to action wield the most pronounced and favorable impact on intention. This substantiates the notion that the broader environmental factors, encompassing interpersonal relationships, advertising campaigns, and ease of accessibility, collectively impinge upon users’ attraction and intention. This empirical finding aligns seamlessly with the observations of Razmara et al. [28] and Yuen et al. [44], whose research underscores the societal and interpersonal influences that significantly mold individual behavior. To elucidate, when individuals within one’s social sphere exhibit specific behaviors, a ripple effect often ensues, with others emulating such behaviors [64,65]. Exploring the consequences of the SERVQUAL analysis (**H7**) unveils a dual impact. The investigation not only exposes an indirect effect of SERVQUAL on intention, mediated by the components of the HBM, but also identifies a direct effect. This twofold influence highlights the enduring significance of comprehensive service quality in cultivating passenger inclination towards embracing railway transportation in the post-pandemic era [66,67]. Hence, enhancing the quality of rail transport services across all facets—be it accessibility, passenger assistance, staff interactions, safety measures, reliability, or other pertinent factors—consequently leads to an increased inclination among passengers to utilize the service. Emanating from the exploration of perceived benefits (**H8**), a constructive impact on intention becomes evident. This prevailing pattern serves as an overarching framework that readily explicates the mechanisms through which users are drawn to a given service. Should railway passengers glean a perception of service quality surpassing the associated costs, their willingness to opt for rail transportation heightens across diverse scenarios. Equally pivotal is the element of health (**H9**), which emerges as a compelling motivation underpinning passengers’ service quality perception. This inference resonates harmoniously with the findings of Ortiz-Sánchez et al. [68], who underscore the potency of health awareness and motivation in shaping attitudes towards activities, ultimately culminating in decision-making dynamics.

On the contrasting side, within the context of this study, certain factors have emerged that exert a negative impact on passengers’ intentions to engage with railway services. These factors encompass perceived susceptibility (**H11**), perceived severity (**H12**), and perceived barrier (**H13**). The findings concerning perceived susceptibility align with the investigations of Morowatisharifabad [29]. This line of reasoning emanates from the recognition that any semblance of sensitivity, vulnerability, or apprehension regarding unforeseen incidents or potential pandemics can arise at any moment. Particularly for passengers who must endure extended periods of travel until reaching their destination terminal, heightened vulnerability is concomitant with a diminished inclination to utilize the service. The perception of risk assumes pivotal importance from the passengers’ perspective, thereby warranting acknowledgment by relevant regulatory bodies. Likewise, perceived severity demonstrates a connection with intention. Passengers who harbor concerns encompassing general safety or the potential outbreak of an epidemic tend to exhibit an acute awareness of the corresponding severity. This observation echoes the findings of Lajunen and Räsänen [43], who discerned that bolstering railway safety engenders heightened passenger confidence, subsequently amplifying their intention to avail railway services. Regrettably, apprehensions regarding change or perceived barriers likewise manifest as deterrents to passenger intention, a phenomenon also corroborated by Razmara et al. [28]. Essentially, this signifies that an escalation in perceived barriers among passengers is directly proportional to a decrease in their intention to patronize rail services. Human behavior often gravitates towards establishing routines for recurrent activities and can be inclined against deviations in the environment. Consequently, unanticipated events or epidemics can elicit anxiety and a sense of insecurity among passengers, thereby undermining their willingness to employ the service. Hence, if service providers aspire to allure and cultivate trust among users, they must consistently refine service quality to ameliorate the barriers residing within passengers’ perceptions.

## 5. Conclusions and Recommendations

This study aimed to assess users’ needs and expectations regarding rail transport services in the post-pandemic context following the widespread pandemic. By examining the interaction between SERVQUAL and the HBM, the study sought to evaluate the general service infrastructure requirements perceived by users and their perspective on health issues (HBM). Furthermore, the study explored how these factors contribute to users’ intentions to utilize rail transportation. To gather data, a face-to-face interview questionnaire was administered to 1600 rail transport users across Thailand. The results of this research indicated that the components of the SERVQUAL model positively influence the HBM, while elements of the HBM impact users’ intentions to use rail transportation. In the preliminary findings, CFA confirmed that all observable variables (questionnaire items obtained from rail users; 20 indicators of SERVQUAL and 28 indicators of HBM) serve as legitimate measures of both SERVQUAL and HBM components. Additionally, these parameters exhibited a strong association with users’ intentions to employ railway transit.

The findings highlighted the continued significance of essential service quality variables (tangibility, reliability, responsiveness, assurance, and empathy) in ensuring satisfactory rail transportation service quality, even after the major epidemic. Moreover, the study revealed that the HBM plays a crucial role in understanding users’ needs. Given the global concern about health issues during the COVID-19 pandemic, it is essential to acknowledge the impact of health awareness on transportation users worldwide [8,10]. Consequently, the inclusion of the HBM in the analysis, comprising perceived susceptibility, perceived severity, perceived benefits, perceived barriers, health motivation, and cue to action, provided valuable insights into users’ perspectives on health issues and their intentions to use railway transportation.

The findings of this study have practical implications for maintaining and improving the quality of rail service. Specifically, the results indicate that relevant agencies (such as State Railway of Thailand (SRT) or Ministry of Transport (MOT), etc.) should strive to keep up their performance in delivering important rail service quality (SERVQUAL) indicators, especially the tangibility of service. The CFA results demonstrated that tangibility represented the most influential factor in rail users’ minds. Significantly, in accordance with this factor, these agencies should take notes on the importance of clear and correct communications from their employees, on-time service, and the cleanliness of facilities etc., followed by empathy, responsiveness, reliability, and assurance, especially. This confirms the importance of the basic elements identified in the SERVQUAL concept (by Parasuraman et al. [32]) for enhancing service quality in rail transport. Moreover, the study investigated the association between the HBM and passengers’ intentions to use rail transportation. Based on the results, safety and health-related authorities, such as the Ministry of Public Health, play a crucial role in developing measures to ensure the health and safety of public transport users. In particular, the evidence suggested that intercity rail users tend to be more health-conscious after the COVID-19 epidemic. Overall, these findings emphasized the need for sustained efforts to maintain and improve the quality of rail service while addressing the health concerns of passengers. By implementing effective measures and involving relevant stakeholders, the rail industry can continue to provide reliable and safe transportation for customers. The interaction between SERVQUAL and HBM in this study demonstrated some important insight: in the context of any research field, if we can include more than one theory (or concept model) in the analysis, it can help us reveal the complexity in respondents’ views. Further study is suggested to add the application of the combination method for exploring the more significant results.

One limitation of our study is its focus on traditional land transport services, particularly intercity trains; therefore, we cannot generalize our findings to other modes of transportation. It is possible that users of different transport modes have varying perspectives and attitudes towards the overall transport system of the country. Future studies could address this limitation by including a broader range of transport modes to provide a more comprehensive picture of user perceptions and preferences. Moreover, conducting similar studies across multiple developing nations may yield valuable insights into the factors shaping public perceptions of transportation services. Such cross-cultural analyses would enable policymakers and practitioners to identify common trends and challenges in the transport sector that transcend national boundaries. Furthermore, it would allow for comparative assessments of the effectiveness of different policies and interventions in various socio-economic contexts.

## Figures and Tables

**Table 1:** The distribution of respondent characteristics.

Code	Definition	1600 Respondents	
		Frequency	%
Gender	Male	827	51.7
	Female	773	48.3
Age	≤25 years	493	30.8
	26–35 years	470	29.4
	36–45 years	385	24.1
	≥46 years	252	15.8
Marital status	Single	796	49.8
	Married	667	41.7
	Otherwise	137	8.6
Education	Below high school	279	17.4
	High school/Uneducated	608	38.0
	Bachelor	649	40.5
	Above Bachelor	64	4.1
Occupation	Student	350	21.9
	Government/State enterprise officer	195	12.2
	Private company	394	24.6
	Self-employed	316	19.8
	Farmer	97	6.1
	Laborer	225	14.1
	Others	23	1.3
Personal income *	Less than 10,000	549	34.3
	10,000–19,999	573	35.8
	20,000–29,999	350	21.9
	30,000 or higher	128	8.0

Note: * Baht per month.

**Table 3:** Descriptive statistics.

Items	Mean	SD	SK	KU	Cronbach’s Alpha
SERVQUAL					
Tangibility					0.875
TAN1	4.37	1.49	0.19	−0.81	
TAN2	4.33	1.53	0.21	−0.75	
TAN3	4.25	1.48	0.26	−0.62	
TAN4	4.80	1.45	0.20	−1.17	
Reliability					0.875
REL1	4.23	1.43	−0.08	−0.34	
REL2	4.48	1.4	0.00	−0.47	
REL3	4.44	1.35	0.12	−0.41	
REL4	3.90	1.54	0.45	−0.42	
Responsiveness					0.882
RES1	4.98	1.07	0.15	0.07	
RES2	4.88	1.06	0.23	0.07	
RES3	4.86	1.08	0.20	−0.01	
RES4	4.92	1.07	0.18	0.28	
Assurance					0.8630
ASS1	4.82	1.20	0.10	−0.55	
ASS2	4.92	1.15	0.17	−0.29	
ASS3	4.9	1.07	0.12	0.15	
ASS4	4.89	1.06	0.21	0.18	
Empathy					0.789
EMP1	4.88	1.29	−0.06	−0.85	
EMP2	4.77	1.30	0.02	−0.71	
EMP3	4.87	1.26	0.01	−0.73	
EMP4	4.82	1.23	0.08	−0.83	
Health belief model					
Intention					0.876
INT1	4.84	1.06	−0.15	0.68	
INT2	4.70	1.02	−0.09	0.77	
INT3	4.77	1.04	0.11	0.56	
INT4	4.73	1.07	0.00	0.53	
Perceived Susceptibility					0.859
PSU1	4.30	1.32	−0.04	−0.36	
PSU2	4.17	1.37	0.030	−0.36	
PSU3	4.20	1.36	−0.05	−0.17	
PSU4	4.25	1.35	−0.01	−0.35	
Perceived Severity					0.883
PSE1	4.99	1.10	0.11	−0.03	
PSE2	4.98	1.11	0.12	−0.17	
PSE3	5.00	1.14	0.16	−0.26	
PSE4	5.05	1.14	−0.08	0.00	
PSE5	4.94	1.11	0.15	0.25	
Perceived Benefits					0.841
PBE1	4.28	1.33	0.15	−0.33	
PBE2	4.34	1.33	0.11	−0.39	
PBE3	4.29	1.30	0.14	−0.24	
PBE4	4.06	1.40	−0.12	−0.17	
Perceived Barriers					0.880
PBA1	3.56	1.68	0.25	−0.73	
PBA2	3.31	1.67	0.30	−0.76	
PBA3	3.89	1.52	0.34	−0.54	
PBA4	3.82	1.47	0.49	−0.39	
Cues to Action					0.824
CUE1	4.63	1.11	−0.08	0.71	
CUE2	4.69	1.10	−0.05	0.63	
CUE3	4.59	1.16	−0.09	0.42	
CUE4	4.55	1.15	−0.33	1.02	
Health Motivation					0.726
MOT1	4.89	1.29	0.10	−0.81	
MOT2	4.68	1.23	0.09	−0.42	
MOT3	4.87	1.22	0.14	−0.75	

Note: Sample size = 1600; SD = standard deviation; SK = skewness; KU = kurtosis.

**Table 4:** Standardized model results (confirmatory factor analysis).

Health Belief Model				SERVQUAL			
Items	Standardized Coefficient	S.E.	*p*-Value	Items	Standardized Coefficient	S.E.	*p*-Value
Intention (0.628) [0.995]				Tangibility (0.650) [0.996]			
INT1	0.736	0.013	<0.001	TAN1	0.862	0.008	<0.001
INT2	0.803	0.013	<0.001	TAN2	0.869	0.008	<0.001
INT3	0.832	0.011	<0.001	TAN3	0.88	0.007	<0.001
INT4	0.796	0.012	<0.001	TAN4	0.572	0.018	<0.001
Perceived susceptibility (0.574) [0.994]				Reliability (0.669) [0.996]			
PSU1	0.836	0.01	<0.001	REL1	0.749	0.012	<0.001
PSU2	0.706	0.015	<0.001	REL2	0.873	0.008	<0.001
PSU3	0.686	0.016	<0.001	REL3	0.889	0.007	<0.001
PSU4	0.793	0.012	<0.001	REL4	0.751	0.012	<0.001
Perceived severity (0.624) [0.996]				Responsiveness (0.639) [0.996]			
PSE1	0.735	0.014	<0.001	RES1	0.835	0.009	<0.001
PSE2	0.746	0.014	<0.001	RES2	0.767	0.012	<0.001
PSE3	0.828	0.011	<0.001	RES3	0.776	0.012	<0.001
PSE4	0.764	0.012	<0.001	RES4	0.817	0.01	<0.001
PSE5	0.867	0.013	<0.001				
Perceived benefit (0.574) [0.994]				Assurance (0.639) [0.996]			
PBE1	0.84	0.01	<0.001	ASS1	0.709	0.015	<0.001
PBE2	0.853	0.009	<0.001	ASS2	0.821	0.009	<0.001
PBE3	0.752	0.013	<0.001	ASS3	0.845	0.009	<0.001
PBE4	0.545	0.019	<0.001	ASS4	0.815	0.01	<0.001
Perceived barriers (0.706) [0.994]				Empathy (0.504) [0.992]			
PBA1	0.79	0.018	<0.001	EMP1	0.772	0.014	<0.001
PBA2	0.61	0.018	<0.001	EMP2	0.602	0.018	<0.001
PBA3	0.969	0.016	<0.001	EMP3	0.745	0.013	<0.001
PBA4	0.942	0.016	<0.001	EMP4	0.708	0.016	<0.001
Cue to action (0.513) [0.992]				Health motivation (0.472) [0.987]			
CUE1	0.665	0.017	<0.001	MOT1	0.743	0.017	<0.001
CUE2	0.813	0.012	<0.001	MOT2	0.593	0.019	<0.001
CUE3	0.757	0.014	<0.001	MOT3	0.715	0.018	<0.001
CUE4	0.614	0.019	<0.001				

Note: AVE showed in (parentheses), and CR showed in [brackets]; Model *(HBM)*: $\chi^{2}/df$ = 2.764, CFI = 0.981, TLI = 0.975, SRMR = 0.032, and RMSEA = 0.028; Model *(SERVQUAL)*: $\chi^{2}/df$ = 3.221, CFI = 0.986, TLI = 0.980, SRMR = 0.028, and RMSEA = 0.037.

**Table 5:** Correlations between constructs and discriminant validity.

	INT	PSU	PSE	PBE	PBA	CUE	MOT	TAN	REL	RES	ASS	EMP
INT	**0.792**											
PSU	0.481	**0.758**										
PSE	0.432	0.435	**0.790**									
PSE	0.463	0.635	0.325	**0.758**								
PBA	0.382	0.568	0.480	0.493	**0.840**							
CUE	0.566	0.446	0.429	0.508	0.449	**0.716**						
MOT	0.475	0.504	0.557	0.473	0.487	0.407	**0.687**					
TAN	0.467	0.625	0.474	0.554	0.622	0.355	0.602	**0.806**				
REL	0.431	0.604	0.373	0.604	0.517	0.278	0.548	0.755	**0.817**			
RES	0.541	0.439	0.557	0.476	0.483	0.410	0.568	0.623	0.619	**0.799**		
ASS	0.583	0.471	0.608	0.445	0.536	0.439	0.594	0.654	0.599	0.781	**0.799**	
EMP	0.479	0.470	0.389	0.503	0.308	0.310	0.504	0.547	0.614	0.643	0.618	**0.710**

Note: All indicators were significant at the 0.01 level (2-tailed); square roots of AVE are presented in bold in the diagonal row.

**Table 6:** Factors influencing the passengers’ intentions (structural equation modeling).

Hypothesis	Variable	Standardized Coefficient	*t*-Value	*p*-Value	Results
Measurement model:					
	SERVQUAL measured by;				
	Tangibility	0.943	0.011	<0.001	Supported
	Reliability	0.818	0.011	<0.001	Supported
	Responsiveness	0.843	0.012	<0.001	Supported
	Assurance	0.804	0.012	<0.001	Supported
	Empathy	0.891	0.013	<0.001	Supported
Structural model:					
	SERVQUAL effect on;				
H1	Perceived benefits	0.795	0.017	<0.001	Supported
H2	Health motivation	0.842	0.017	<0.001	Supported
H3	Cue to action	0.468	0.024	<0.001	Supported
H4	Perceived susceptibility	−0.222	0.007	<0.001	Supported
H5	Perceived severity	−0.298	0.012	<0.001	Supported
H6	Perceived barriers	−0.183	0.007	<0.001	Supported
	Intention affected by;				
H7	SERVQUAL	0.460	0.017	<0.001	Supported
H8	Perceived benefits	0.284	0.01	<0.001	Supported
H9	Health motivation	0.242	0.01	<0.001	Supported
H10	Cues to action	0.463	0.019	<0.001	Supported
H11	Perceived susceptibility	−0.276	0.01	<0.001	Supported
H12	Perceived severity	−0.206	0.009	<0.001	Supported
H13	Perceived barriers	−0.503	0.021	<0.001	Supported

## Data Availability

Data are available on request due to privacy restrictions.
